# Supplementary material for: Oregano (Origanum vulgare) Essential Oil and Its Constituents Prevent Rat Kidney Tissue Injury and Inflammation Induced by a High Dose of L-Arginine
Source: Int J Mol Sci. 2024 Jan 11;25(2):941. doi: 10.3390/ijms25020941 (PMC10815453; doi:10.3390/ijms25020941)
Supplement: Supplementary file 1 [file ijms-25-00941-s001.zip › ijms-2801607-supplementary.pdf]

Table S1. Chemical composition of *O. minutiflorum* essential oil

RI [a]	RI [b]	Constituent [c]	Content [d]	Class [e]
785	776	Methyl 2-methylbutanoate	0.1	O
929	924	α-Thujene	0.6	M
937	932	α-Pinene [f]	0.5	M
954	946	Camphene [f]	0.2	M
977	974	1-Octen-3-ol [f]	0.1	O
983	974	β-Pinene[f]	0.2	M
992	988	Myrcene [f]	0.7	M
995	988	3-Octanol [f]	Tr [h]	O
1009	1002	α-Phellandrene [f]	0.1	M
1015	1008	Δ^3-Carene	tr	M
1020	1014	α-Terpinene [f]	0.8	M
1028	1020	*p*-Cymene [f]	3.9	M
1032	1024	Limonene [f]	0.3	M
1032	1025	β-Phellandrene [f]	tr	M
1035	1026	1,8-Cineole [f]	0.3	MO
1037	1032	(*Z*)-β-Ocimene	tr	M
1048	1044	(*E*)-β-Ocimene	tr	M
1061	1054	γ-Terpinene [f]	3.1	M
1070	1065	*cis*-Sabinene hydrate	0.5	MO
1075	1067	*cis*-Linalool oxide (furanoid) [f]	0.2	MO
1092	1084	*trans*-Linalool oxide (furanoid) [f]	0.3	MO
1102	1095	Linalool [f]	33.1	MO
1098	1107	*trans*-Sabinene hydrate	tr	MO
1126	1118	*cis*-*p*-Menth-2-en-1-ol	tr	MO
1143	1136	*trans*-*p*-Menth-2-en-1-ol	tr	MO
1150	1141	Camphor [f]	tr	MO
1171	1165	Borneol [f]	0.6	MO
1176	1173	*trans*-Linalool oxide (pyranoid)	tr	MO
1182	1174	Terpinen-4-ol [f]	0.4	MO
1189	1179	*p*-Cymen-8-ol	tr	MO
1195	1186	α-Terpineol [f]	0.4	MO
1200	1191	*cis*-Dihydro carvone	tr	MO
1210	1200	*trans*-Dihydro carvone	tr	MO
1246	1241	*O*-Methyl carvacrol [f]	0.2	MO
1250	1239	Carvone [f]	0.1	MO
1284	1277	2,6-Dimethyl-1,7-octadiene-3,6-diol [g]	tr	MO
1287	1289	Isothymol	tr	MO
1294	1289	Thymol [f]	2.5	MO
1298	1307	Carvacrol [f]	46.9	MO
1375	1370	Carvacryl acetate	0.1	MO
1391	1387	β-Bourbonene	tr	S
1426	1417	(*E*)-Caryophyllene [f]	1.9	S
1435	1430	β-Copaene	tr	S
1446	1439	Aromadendrene [f]	0.3	S
1460	1452	α-Humulene [f]	0.1	S
1468	1464	9-*epi*-(*E*)-Caryophyllene	tr	S
1482	1483	α-Amorphene	tr	S
1501	1496	Viridiflorene	tr	S
1503	1500	Bicyclogermacrene	tr	S

1512	1505	*β*-Bisabolene [f]	0.3	S
1520	1513	*γ*-Cadinene	tr	S
1529	1522	*δ*-Cadinene	tr	S
1554	1548	Elemol	tr	SO
1584	1577	Spathulenol [f]	tr	SO
1590	1582	Caryophyllene oxide [f]	0.3	SO
1647	1638	*τ*-Cadinol	0.1	SO
1658	1649	*β*-Eudesmol	tr	SO
1661	1662	7-*epi*-*α*-Eudesmol	tr	SO
**Total identified (%)**			**99.2**	
Monoterpene hydrocarbons (M)			10.4	
Oxygenated monoterpenes (MO)			85.6	
Sesquiterpene hydrocarbons (S)			2.6	
Oxygenated sesquiterpenes (SO)			0.4	
Others (O)			0.2	

[a] Retention indices determined experimentally on a DB-5MS column relative to a series of C_7–C_{40} *n*-alkanes. [b] Literature values of retention indices taken from Adams [1] or NIST [2] collection. [c] Compound identified based on mass spectra and retention indices matching with literature data. [d] Values are means of three individual analyses. [e] For compound class abbreviations, *cf.* last rows of this Table. [f] Compound identity confirmed by a co-injection experiment with a standard. [g] tr, trace amounts (<0.05%). [h] Correct diastereomer not determined.

References

1. R. P. Adams, 'Identification of Essential Oil Components by Gas Chromatography/Mass Spectrometry 4th ed.; Allured Publishing: Carol Stream, Illinois, USA, 2007.
2. NIST Mass Spectrometry Data Center, William E. Wallace, director, "Retention Indices" in NIST Chemistry WebBook, NIST Standard Reference Database Number 69, Eds. P.J. Linstrom and W.G. Mallard, National Institute of Standards and Technology, Gaithersburg MD, 20899, https://doi.org/10.18434/T4D303, (retrieved September 10, 2020).
